# Marine fungi: A treasure trove of novel natural products and for biological discovery

**DOI:** 10.1371/journal.ppat.1011624

**Published:** 2023-09-21

**Authors:** Frank Kempken

**Affiliations:** 1 Abteilung Botanische Genetik und Molekularbiologie, Botanisches Institut und Botanischer Garten, Christian-Albrechts-Universität zu Kiel, Kiel, Germany; University of Maryland, Baltimore, UNITED STATES

## Introduction

Originally being thought to be absent from salt water, compelling evidence was presented for the existence of marine fungi. This research was published as a textbook in1979 [1], but at first, it was not widely recognized in the fungal community. Since that time, a growing number of fungi have been described from marine environments, such as mangroves, beaches, seaweeds, sponges, and seabed, to name a few [2–4]. Because of the chilotrophic lifestyle of fungi, which includes the external digestion of food sources by secreted enzymes followed by the uptake of low molecular substances, it had been questioned whether fungi can dwell in open seawater. According to new studies, it is now clear that pelagic fungi are ubiquitous in open sea waters and play an active role in organic matter degradation and nutrient cycling [5].

There is a strong correlation between fungi sampled in deep sea environments and clades of known terrestrial fungi. It thus appears that fungi that live in surface environments on land can easily adapt to marine environments. Every year, fungi release millions of tons of fungal spores into the atmosphere [6], which may end up in marine environments, but are not true marine fungi in the sense that they permanently live and propagate in marine environments. While this makes it difficult to define a marine fungus, in general, most if not all fungi seem well suited to live in marine habitats, if not dwelling there, which is due to their ability to tolerate high salt concentrations and to withstand high hydrostatic pressure. During short-term experiments, it became evident that fungi can alter their membrane composition, allowing them to withstand high hydrostatic pressure [7]. Adaption to high pressure may, however, require other specific adaptations, the nature of which remains largely unknown. Hence, taking these aspects into account, in many cases, it may be best to talk about marine adaption of fungi or marine-derived fungi rather than marine species, although some marine-derived fungi do require high salt concentration for growth and seem very much dependent on marine conditions. This, however, led to the question what strategies fungi have developed to scope with high salt stress. A major response is the utilization of compatible solutes such as glycerol in the cytosol to keep internal Na^+^ ion level low. In addition, cell wall structure and melanization are associated with salinity stress. The melanization of cell walls has a role in effective intracellular retention of glycerol. Thirdly, maintenance of ion homeostasis using metal cation transporters may be a salt adaptation strategy [8].

**Fig 1:**
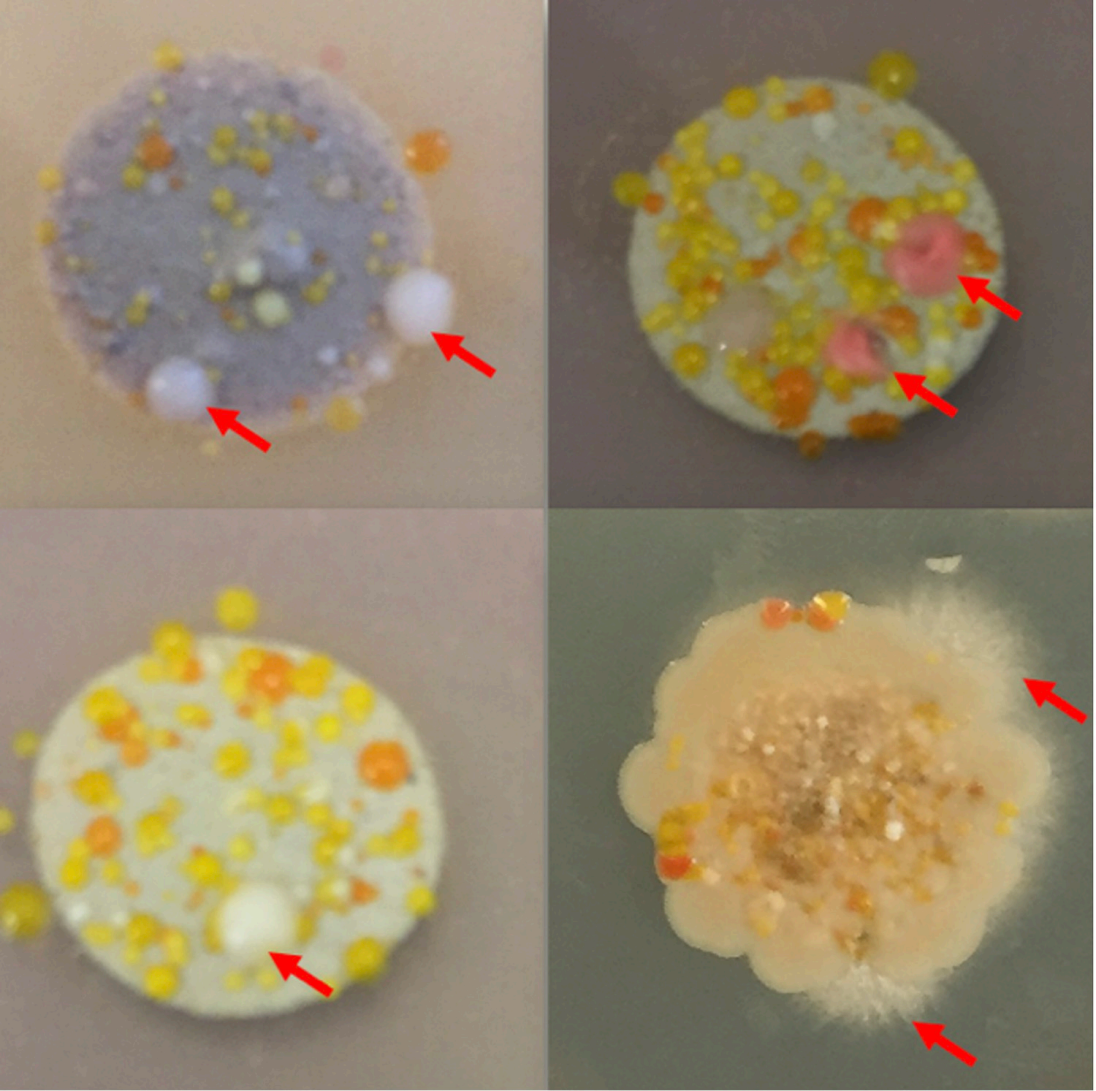
Isolation of fungi from mid-Atlantic Ridge seabed. Seabed samples from different depth were mixed with 0.9% NaCl solution, and 50 μl of each were plated on salt (30 g NaCl/L) containing medium with streptomycin to reduce bacterial growth and kept at 25°C for 14 days [8]. Arrows indicate yeast-like colonies or mycelial fungi. Other colonies resemble streptomycin-resistant bacteria. *Picture*: *F*. *Kempken*.

Some fungi not only had been detected as DNA sequences in environmental samples from ocean seabed [2] but also have been isolated and cultured from seabed sampled at thousands of meters depth (see Fig 1). Among these was a strain of the red yeast *Rhodotorula mucilaginosa*, which was analyzed for its biosynthetic potential. Such red yeasts are of interest to industry due to their ability to produce valuable natural products, such as lipids and carotenoids with potential applications as surfactants, food additives, and pharmaceuticals [8].

Depending on the culture medium, the marine *R*. *mucilaginosa* strain exhibited anticancer activity or antimicrobial activity. Applying a bioactive molecular networking approach, the anticancer activity was linked to glycolipids, namely, polyol esters of fatty acid derivatives (PEFA). Further investigations by Illumina-based genome sequencing, de novo assembly and standard biosynthetic gene cluster analyses illustrated key components of the PEFA biosynthetic pathway [9].

Often similar fungal strains or species are being identified from marine and terrestrial sources. Within the EU framework Marine Fungi, the genome of a strain of *Scopulariopsis brevicaulis* was sequenced [10], which was isolated from the marine sponge *Tethya aurantium*. It produces 2 novel cyclodepsipeptides, scopularides A and B with anticancer properties [11]. Originally described in 1882, *S*. *brevicaulis* became known as the *Arsenic Fungus* because in the presence of carbohydrates, the fungus can degrade arsenic-containing paints, such as Schweinfurt green (copper arsenite acetate), to form gaseous, toxic trimethylarsine. In 1998, *Microascus brevicaulis* was discovered as the teleomorph or sexual stage of *S*. *brevicaulis*. Interestingly, onychomycosis is a condition caused by this pathogenic fungus [12]. To sum up, *S*. *bevicaulis* may be a nail fungus, a sponge inhabitant or was known to dwell in moist wallpaper. Accordingly, one may wonder how similar marine and terrestrial strains of the same fungal species may be. Unfortunately, there are no comparative studies of the genomes of different *S*. *brevicaulis* strains available, which would allow to elucidate the true relations between marine and terrestrial strains.

In this review, certain aspects of marine fungi will be discussed, with an emphasis on secondary metabolite production and carbon degradation, as this may be an important ecological function of marine-associated fungi. Likewise, hydrophobins are included due to their practical applications and a potential role in salt adaptation.

## Secondary metabolite gene cluster

Different methods can be used to identify secondary metabolite gene clusters in genomic sequences [13–15]. The number and type of secondary metabolite gene clusters found in fungi, particularly filamentous ascomycetes, varies largely. There is a high degree of diversity in secondary metabolite gene clusters, and even closely related fungal strains may possess different secondary metabolites. For example, in the 46-Mb genome of the marine strain *Pestalotiopsis* sp. KF079 isolated from the North Sea mudflats, there are 67 secondary metabolite gene clusters. These clusters include 22 PKS clusters, 12 nonribosomal peptide synthetase clusters, 9 terpene clusters, and 2 other clusters. Additionally, *Pestalotiopsis* sp. KF079 has 6 clusters of hybrid nature as well as a number of putative uncharacterized clusters [16]. Out of 67 secondary metabolite gene clusters of *Pestalotiopsis* sp., 65 were found to be previously uncharacterized.

Marine-derived fungi contain a wealth of secondary metabolites, many of which are bioactive or pharmaceutically valuable [3,17]. In a recent review, the biotechnological value of secondary metabolites is emphasized, including the description of 187 new compounds and 212 others with anticancer and antibacterial properties [3]. Hence, here is great potential for biotechnological use of these [17]. It further appears that the molecular diversity of the secondary metabolites of marine fungal isolates reflects their immense biodiversity. So far, less than 2,000 marine-associated fungi have been described, which are of different phylogentic groups, including Aphelidiomycota, Ascomycota, Basidiomycota, Blastocladiomycota, Chytridiomycota, Mortierellomycota, and Mucoromycota [4]. However, it is believed that the true number of marine-adapted fungi is much higher. Estimates in a recent review [4] range from 10,000 to more than 1 million marine-derived fungal species.

Under laboratory conditions, most of the secondary metabolite gene clusters are transcriptional silent and not expressed, which is a great challenge regarding their biotechnological usability. For example, there were 16 nonribosomal peptide synthase (NRPS) genes identified in the genome of a marine-derived strain of *S*. *brevicaulis*, but only 9 showed significant expression and only 1 polyketide synthase (PKS) gene showed significant expression [10]. The effects were even more profound in 2 other marine-derived fungal strains from the North Sea mudflats: 4 out of 32 NRPS genes in *Calcarisporium* sp. strain KF525 and 4 out of 18 NRPS genes from *Pestalotiopsis* sp. strain KF079 were found to be expressed. Of the 36 PKS genes in *Pestalotiopsis* sp. strain KF079, only 2 showed significant and 9 weak expression [16] in the laboratory. In contrast to laboratory conditions, a wide variety of species are encountered by fungi in their different marine habitats, including bacteria, other fungi, worms, sponges, vertebrates, etc. A groundbreaking study by Brackhage and colleagues [18] found that specific interactions between fungi and bacteria could activate certain secondary metabolite clusters. Similar effects were reported from interactions between fungi and insects [19].

Over the years, many different methods have been established to induce transcriptional silent gene clusters, including the addition of a physical scaffold, addition of small molecule elicitors, and cocultivation with another microbe [20]. Yet, another often fruitful approach is heterologous expression of SM gene clusters in a suitable heterologous host, which may require certain genetic manipulations of the SM gene clusters [21]. Histone methylation also may be responsible for secondary metabolite gene cluster repression [22]. Therefore, analyzing the effect of chromatin methylation on SM gene clusters would be extremely useful. By combining immunoprecipitation with high-throughput ChIP-seq, antibodies against dimethylated H3K4, trimethylated H3K9, and trimethylated H3K27 euchromatin, obligate heterochromatin, and facultative heterochromatin could be identified under marine and laboratory conditions. Using this information, one could determine whether chromatin methylation affects secondary metabolite gene cluster expression in marine fungi. These and other activation techniques, such as coculture, precursor feeding, or altered fermentation procedures, have been reviewed recently [23]. Other strategies include a combination of the ecological role of marine-derived fungi and the design of access routes for biotechnological production, which, however, would require a multidisciplinary approach [24].

## Hydrophobins

Hydrophobin are small (20 kDa) secreted hydrophobic fungi-specific cell wall proteins involved in the formation of aerial structures (such as spores and fruiting bodies) [25]. Hydrophobins possess an amphiophilic structure that determines their self-assembly at hydrophilic–hydrophobic interfaces and surfactant properties, which are useful in biotechnological applications. The marine fungus *S*. *brevicaulis* LF580 contains 3 hydrophobin genes and *Pestalotiopsis* sp. and *Calcarisporium* sp. genomes were found to encode 12 and 2 hydrophobins, respectively [10,17]. Additionally, previous analyses of marine fungi found variable numbers of hydrophobin genes, which, however, may be regulated by salt stress adaptations. In a screening of 100 marine fungi, they were found to be a fresh source of hydrophobins [25].

## Carbohydrate-active enzymes

There is limited understanding of the lifestyle and ecological importance of marine fungi. A low-nutrient environment favors organisms that feed primarily by attaching to larger physical substrates and by osmotrophy. Carbon fixation by plants and seaweeds either on land or in marine environments provides a large portion of the energy and nutrients. The digestion of these plant tissues require carbohydrate-active enzymes (CAZ), which have various applications in biotechnology [26]. Using the CAZy database, 949 and 476 CAZ genes were identified in 2 marine-derived strains (Pestalotiopsis and Calcarisporium) from the North Sea mudflats. Six major classes of CAZ, which include 423 glycoside hydrolases (GH), 122 glycosyltransferases (GT), 35 polysaccharide lyases (PL), 80 carbohydrate esterases (CE), 134 carbohydrate binding modules (CBM), and 155 auxiliary activities (AA), have been identified in Pestalotiopsis [16]. These numbers are not radically different from those of terrestrial fungi, indicating similar ecological roles and a certain dependence on plant carbohydrates for nutrition. In comparison, CAZ candidates of *Calcarisporium* sp. are radically lower in number for each class except for GT. Similar data were found in *S*. *brevicaulis* strain LF581 [9]. CAZ genes from pelagic fungi have been reported to have high transcriptional activity in carbohydrate degradation [27]. In their study, Dothideomycetes in epipelagic waters and Leotiomycetes in mesopelagic waters are the 2 main pelagic fungi responsible for carbohydrate degradation. In addition to CAZ, pelagic fungi digest proteins and appear to play diverse ecological roles [28,29]. These data indicate that marine fungi do not generally differ from terrestrial species [4,17].

## Conclusions

In spite of being almost ignored for years, marine fungi are now an exciting area of fungal research. Regardless of the ongoing debate over the definition of marine fungi, it has become evident that they are present in nearly all, if not all, marine ecological niches, where they play an important role in carbohydrate degradation and nutrient cycling requiring further attention. A plethora of new secondary metabolites, many of which are highly useful in biotechnology, are produced by marine fungi, which we are only beginning to appreciate. Unfortunately, identifying new secondary metabolites is still hampered by the repression of secondary metabolite genes in marine fungi, which may be overcome by coculturing with marine bacteria, other marine fungi, or by manipulating histone methylation. Future work on marine fungi will help answer these questions and represent a highly attractive field of research.
